# A narrative review of stem cell-based therapy for fibrosing interstitial lung diseases: clinical evidence and translational perspectives

**DOI:** 10.3389/fphar.2026.1917005

**Published:** 2026-10-07

**Authors:** Zhengnan Li, Lei Geng, Wei Li, Xiujuan Wang, Xiu’e Wang, Zhen Qin, Ya'nan Zhang, Chunlin Mou

**Affiliations:** Research and Development Center, Tianjin Huayu Pharmaceutical Co., Ltd., Tianjin, China

**Keywords:** delivery routine, fibrosing interstitial lung diseases, idiopathic pulmonary fibrosis, mesenchymal stem cells, post COVID-19 pulmonary fibrosis

## Abstract

Fibrosing interstitial lung diseases (F-ILD) are a heterogeneous group of disorders characterized by pulmonary interstitial fibrosis and inflammation. Idiopathic pulmonary fibrosis (IPF) is the most common and severe form of F-ILD. Regulator-approved anti-fibrotic drugs, such as pirfenidone and nintedanib, delay rather than reverse disease progression, so the threat of eventual respiratory failure remains. While lung transplantation is an option that can prolong survival for end-stage IPF, its clinical application is greatly restricted by donor shortages, surgical risks, and postoperative complications. Stem cell therapies, and particularly treatments based on mesenchymal stromal/stem cells (MSCs) and their derivatives, have brought new hope for the management of F-ILD due to their immune regulatory, anti-inflammatory, and anti-fibrotic capabilities. A search was conducted to identify relevant articles in the PubMed/MEDLINE, Web of Science, Cochrane Library, China National Knowledge Internet and Wanfang Data from inception to 31st May 2026. This review summarizes the mechanistic overview of stem cell-based therapeutic strategies for F-ILD, safety profiles and therapeutic potentials of various stem cells for the treatment of IPF and other F-ILDs, with an emphasis on stem cell types and delivery routine. This article also discusses the challenges and limitations of stem cell-based therapies, highlighting the need for further clinical studies with standardized efficacy endpoints to validate their effectiveness.

## 1 Introduction

Fibrosing interstitial lung diseases (F-ILDs) are a heterogeneous group of disorders characterized by pulmonary interstitial fibrosis and inflammation. Idiopathic pulmonary fibrosis (IPF), the most common and severe F-ILD, is a chronic, progressive disorder of unknown etiology that manifests as progressive dyspnea and deterioration of lung function with fibrotic changes in the early stage. Clinically, progressive F-ILDs are characterized by worsening pulmonary fibrosis despite etiological treatment and the administration of conventional therapies such as glucocorticoids and immunosuppressants. In 2022, the updated clinical practice guideline standardized the terminology for progressive F-ILDs other than IPF and proposed the concept of progressive pulmonary fibrosis (PPF) (Raghu et al., 2022). Epidemiologic evidence indicates that the incidence of IPF is increasing worldwide and that the prevalence of IPF is higher in North America than in Europe or Asia (Golchin et al., 2025; Spagnolo et al., 2025). The median survival of patients with IPF is 2–3 years (Ley et al., 2011), although some patients live longer. Antifibrotic agents such as pirfenidone (Noble et al., 2011; King et al., 2014) and nintedanib (Richeldi et al., 2014) have been shown to attenuate the rate of decline in lung function (primarily measured as the forced vital capacity, FVC) among patients with IPF, heralding a new era in disease management since 2011. However, these treatment regimens fail to reverse pre-existing fibrosis, and their clinical efficacy is predominantly confined to delaying disease progression rather than achieving a cure. Moreover, antifibrotic agents exhibit only limited efficacy in a substantial subset of patients and are associated with adverse effects such as gastrointestinal and skin-related reactions (Noble et al., 2011; King et al., 2014; Richeldi et al., 2014). More importantly, the current antifibrotic strategies have been studied mainly in patients with IPF, so there is only limited evidence regarding their efficacy in other F-ILDs such as fibrosing hypersensitivity pneumonitis, idiopathic nonspecific interstitial pneumonia, and connective tissue disease-associated ILD (CTD-ILD) (Finnerty et al., 2021; Li et al., 2024). In contrast to IPF, these other F-ILD subtypes primarily manifest with inflammation at the early stage and exhibit distinct risk factors for subsequent progression to PPF depending on the underlying etiology (Raghu et al., 2022). The emergence of post-infection fibrosis, such as long COVID-related pulmonary fibrosis, has presented additional challenges such as limited treatment options and inadequate response to immunosuppressive therapy in some patients (Duong-Quy et al., 2025). While lung transplantation is a therapeutic option for end-stage IPF that can improve survival (Thabut et al., 2003), its clinical application is greatly restricted by donor shortages, surgical risks, and postoperative complications. Therefore, there is an urgent clinical need for novel treatment strategies that are capable of repairing damaged lung tissues, regulating abnormal immune responses, and to reverse the fibrotic process.

Against this backdrop, regenerative medicine strategies, especially stem cell-based therapies using mesenchymal stromal/stem cells (MSCs) and their derivatives, have been rapidly transitioned from laboratory research to clinical trials over the past decade (Wei et al., 2013). In contrast to antifibrotic pharmacological therapies that aim to slow disease progression, stem cell-based therapy is designed to repair damaged lung tissues and reverse the fibrotic process throughout the homing, differentiation, and paracrine properties of stem cells (Inamdar and Inamdar, 2013; Harrell et al., 2019; Behnke et al., 2020). The research focus has gradually shifted from early-stage concept validation and safety evaluation to exploration of treatment efficacy across different F-ILD subtypes, stem cell sources, and delivery routes (Ghadiri et al., 2016; Macagno et al., 2017; Ji et al., 2020; Sieiro Santos and Del Galdo, 2025; Yuan et al., 2025). However, stem cell-based therapy still faces numerous challenges, including determination of the optimal cell types, dosages, treatment timing, and the delivery route, identification for the patient subgroups most likely to derive benefit, and standardization of the design of efficacy endpoints.

This review summarizes recent clinical evidence and translational advances regarding stem cell-based therapy for different types of F-ILD, with a particular focus on safety and efficacy outcomes from published human studies, thereby providing insights for future clinical practice and research.

## 2 Methods

### 2.1 Databases and search strategy

This narrative review focuses on clinical evidence and translational research regarding stem cell-based therapy for F-ILD, with evidence sources primarily including published systematic reviews, meta-analyses, observational studies, and clinical trials in Chinese or English. A two-tier literature search was initiated in May 2026. The primary search focused on human clinical studies of stem cell-based therapy, using PubMed/MEDLINE, Web of Science, Cochrane Library, China National Knowledge Internet (CNKI) and Wanfang Data from inception to 31st May 2026. Additionally, the reference lists of the included manuscripts were systematically screened to identify additional relevant studies. Search terms included (“mesenchymal stem cell” OR “mesenchymal stromal cell” OR “lung progenitor cell” OR “alveolar epithelial type II cell” OR “exosomes” OR “extracellular vesicle”) AND (“idiopathic pulmonary fibrosis” OR “interstitial lung disease” OR “pulmonary fibrosis” OR “connective tissue disease-associated interstitial lung disease” OR “COVID-19 pulmonary fibrosis” OR “long COVID”) AND (“clinical trial” OR “clinical study” OR “case report” OR “safety” OR “efficacy” OR “translational”). A supplementary search was conducted for mechanistic and preclinical studies using terms such as (“mechanism” OR “paracrine” OR “immunomodulation” OR “differentiation” OR “homing” OR “exosome” OR “extracellular vesicle” OR “mitochondrial transfer” OR “microenvironment”) AND (“signaling pathway” OR “*in vitro*” OR “animal model” OR “preclinical study”). In addition, for relevant studies published online after the search cutoff date, those that were published online either prior to the submission of this manuscript or during its revision stage will be treated as supplementary sources of evidence.

### 2.2 Eligibility criteria

Inclusion criteria were: (1) Original research, clinical trials, systematic reviews, and narrative reviews focusing on stem cells or stem cell-derived products for the treatment of pulmonary fibrosis/ILD; (2) Study designs that encompassed randomized controlled trials (RCTs), non-RCTs, single-arm clinical trials, cohort studies, case reports, and preclinical animal model studies; (3) Reporting of clear outcome measures related to safety, efficacy, mechanism of action, or translational potential.

Exclusion criteria were: (1) Stem cell studies not directly relevant to F-ILD treatment; (2) Studies that solely investigated hematopoietic stem cell transplantation (HSCT) for hematological disorders with no reported pulmonary outcomes; (3) Conference abstracts, correspondence, and commentaries (unless critical data were included); (4) Studies consisting solely of *in vitro* experiments without *in vivo* validation (unless directly relevant to mechanistic elucidation); (5) Publications with inaccessible full texts or duplicate publication.

### 2.3 Screening procedure (narrative)

Two independent reviewers screened titles and abstracts, followed by full-text assessment, in accordance with prespecified eligibility criteria. Disagreements were resolved through iterative discussion with a third reviewer until consensus was achieved. Human clinical evidence, particularly data from RCTs, prospective observational studies or other clinical studies was prioritized as the primary source of evidence. For F-ILD subtypes lacking sufficient human clinical data, mechanistic and therapeutic insights derived from *in vitro c*ellular models and *in vivo* animal studies were incorporated to inform translational potential; such evidence was explicitly designated as “preclinical evidence” throughout the synthesis. Mechanistic discussions are primarily integrated with preclinical evidence. Because this is a narrative review, no quantitative synthesis or quantitative bias risk assessment was performed; the screening process is reported narratively. Nevertheless, the sample sizes, stages, endpoints, and safety events of key trials were systematically summarized.

## 3 Mechanistic overview of stem cell-based therapeutic strategies for F-ILD

### 3.1 Mechanisms underlying the pathogenesis and progression of F-ILD

The pathogenesis of pulmonary fibrosis involves complex interplay among diverse cell types and signaling pathways, while the molecular mechanisms underlying IPF have been extensively characterized (Gao et al., 2025). Aberrant repair mechanisms following recurrent alveolar epithelial cell injury drive the pathogenesis and progression of IPF. Specifically, damaged and dysfunctional alveolar epithelial type II (AEII) cells undergo epithelial-mesenchymal transition (EMT), acquiring an interstitial phenotype, while secreting a large number of profibrotic factors. Upon secretion of multiple pro-fibrotic factors, AEII cells drive the activation of quiescent fibroblasts and their subsequent conversion to a myofibroblast phenotype, known as the fibroblast-to-myofibroblast transition (FMT), thereby facilitating excessive extracellular matrix (ECM) deposition and tissue stiffening (Figure 1). Dysfunction of alveolar epithelial cells, particularly that of AEII cells, is recognized as a key upstream mechanism for the occurrence and progression of F-ILD, given that their functional integrity is indispensable for post-injury regeneration and repair (Parimon et al., 2020; Confalonieri et al., 2022). The latest study presents the first multicenter longitudinal analysis of dynamic changes in circulating endothelial compartment and progenitor cells in IPF patients, proposing a hypothesis that endothelial dysfunction may reflect a chronic and potentially secondary pathological process in IPF, rather than being the primary driver of fibrosis (Smadja et al., 2026).

**FIGURE 1:**
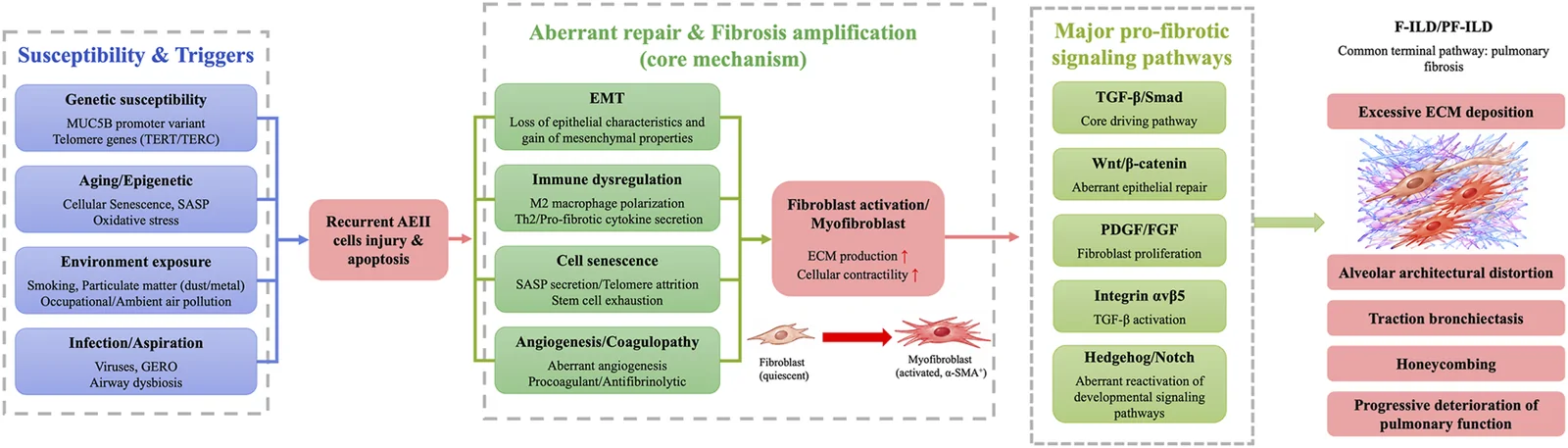
Pathogenesis mechanisms of F-ILD. Abbreviations: AEII, alveolar epithelial type II; ECM, extracellular matrix; EMT, epithelial-mesenchymal transition; FGF, fibroblast growth factor; F-ILD, fibrosing interstitial lung disease; GERD, gastroesophageal reflux disease; MSC, mesenchymal stromal/stem cell; ILD, interstitial lung disease; PDGF, platelet-derived growth factor; PF-ILD, progressive fibrosing-ILD; SASP, senescence-associated secretory phenotype; Smad, Sma and Mad related protein; TERC, telomerase RNA component; TERT, telomerase reverse transcriptase; TGF, transforming growth factor.

The pathogenesis of pulmonary fibrosis is also closely related to stem cell senescence, and IPF has been recognized as an age-associated disease in which telomere attrition and dysfunction constitute critical genetic susceptibility factors (Zhang et al., 2021; Hong et al., 2022). ATII cell-specific telomerase knockout mice exhibited more severe lung injury, inflammation, and pulmonary fibrosis following bleomycin treatment, characterized by impaired AEII cell proliferative capacity and increased cellular senescence (Liu et al., 2019). Cdc42 dysfunction in alveolar stem cells leads to impaired alveolar regeneration, causing persistent elevated mechanical tension on AEII cells, which subsequently activates the TGF-β signaling pathway and drives periphery-to-center progression of pulmonary fibrosis (Wu et al., 2020). In addition, ferroptosis promotes the progression of pulmonary fibrosis, as evidenced by pronounced iron accumulation observed in AEII cells and fibrotic lung tissues of bleomycin-induced fibrosis mice, accompanied by pathological changes, collagen deposition, and ferroptosis in AEII cells (Cheng et al., 2021).

Recent studies have also revealed that lung-resident mesenchymal stem cells (LR-MSCs) can differentiate into myofibroblasts driven by specific microenvironmental signals, thereby constituting a critical source of myofibroblasts during fibrogenesis (Cao et al., 2018; Hou et al., 2018a; 2018b). In the bleomycin-induced pulmonary fibrosis model, TNF-α-mediated NF-κB activation drives the differentiation of LR-MSCs into myofibroblasts (Hou et al., 2018a), whereas Wnt/β-catenin signaling similarly regulates the differentiation of LC-MSCs (Cao et al., 2018). In addition, M2 macrophages promote the differentiation of LR-MSCs into myofibroblasts by activating the Wnt/β-catenin pathway, while macrophage depletion significantly attenuates bleomycin-induced pulmonary fibrosis (Hou et al., 2018b). Endoplasmic reticulum (ER) stress-driven activation of the TGF-β/SMAD pathway, mediated by C/EBP homologous protein (CHOP), facilitates the differentiation of LR-MSCs into myofibroblasts (Yang et al., 2022). Additionally, Sumo specific protein 1 (SENP1)-mediated deSUMOylation dictates the fate of LR-MSCs by stabilizing β-catenin and GLI family zinc finger 1 (GLI1); conversely, silencing SENP1 attenuates myofibroblast differentiation while restoring their regenerative capacity (Sun et al., 2022).

### 3.2 Multiple therapeutic mechanisms of MSCs and other stem cells for F-ILD

MSC-based therapy represents the most extensively investigated strategy for F-ILD (Li et al., 2021). Distinct from single target of small-molecule drugs, MSCs and lung progenitor cells (LPC) exert pleiotropic effects on inflammatory, fibrotic, and epithelial regenerative processes through coordinated mechanisms, including immunomodulation, paracrine action and antifibrotic, extracellular vesicles (EVs)-mediated microRNAs (miRNAs) transfer, mitochondrial transfer, and microenvironment regulation (Figure 2).

**FIGURE 2:**
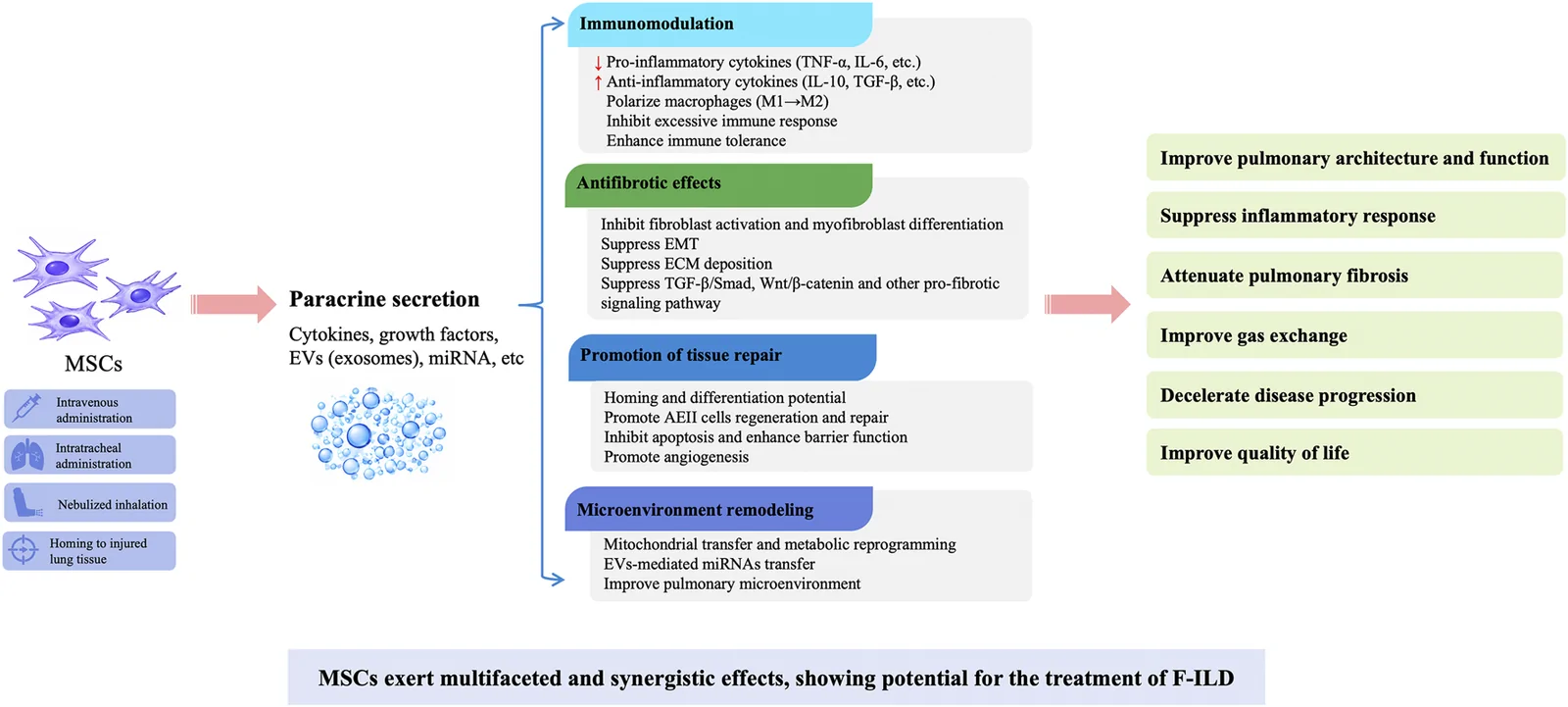
Mechanisms of MSCs therapy in F-ILD Abbreviations: AEII, alveolar epithelial type II; ECM, extracellular matrix; EMT, epithelial-mesenchymal transition; EV, extracellular vesicle; F-ILD, fibrosing interstitial lung disease; IL, Interleukin; MSC, mesenchymal stromal/stem cell; Smad, Sma and Mad related protein; TGF, transforming growth factor; TNF, tumor necrosis factor.

Early studies hypothesized that MSCs repair lung tissue by differentiating into alveolar epithelium following homing and engraftment. Following intravenous administration, MSCs are predominantly entrapped in the pulmonary capillary bed—a phenomenon known as the ‘pulmonary first-pass effect’, and subsequently home to sites of lung injury via mechanisms mediated by the SDF-1/CXCR4 axis, integrins, and selectins. However, *in vivo* tracking studies have revealed that intravenously administered MSCs are predominantly subjected to mechanical entrapment within the pulmonary capillary bed, followed by rapid apoptosis and clearance within days to 2 weeks; consequently, long-term engraftment and differentiation into functional lung parenchymal cells remained negligible (Leibacher et al., 2017). This evidence has driven a paradigm shift in understanding MSC efficacy, moving away from the traditional view of direct cell engraftment toward a model centered on paracrine actions and immunomodulation.

Immunomodulation is one of the core mechanisms underlying MSC therapy for pulmonary fibrosis, wherein MSCs orchestrate innate and adaptive immune responses through the secretion of various soluble mediators. MSCs exert potent immunomodulatory effects by attenuating the proliferation and activation of T, B, and NK cells, facilitating the induction of regulatory T cells (Tregs), and driving macrophage polarization toward an anti-inflammatory phenotype (Ni et al., 2018; Kavianpour et al., 2020). Administration of MSCs-derived conditioned medium also significantly alleviates pulmonary fibrosis by suppressing collagen deposition and attenuating the secretion of pro-inflammatory or pro-fibrotic mediators, including transforming growth factor (TGF)-β1 and interleukin (IL)-6 (Ali et al., 2020). Furthermore, MSCs exert their antifibrotic effects primarily through paracrine actions involving the secretion of various soluble mediators that modulate pulmonary repair processes, among which hepatocyte growth factor (HGF) plays a crucial role (Cahill et al., 2016). Notably, bone marrow MSCs (BM-MSCs) exert antifibrotic effects in silicosis models through a multi-pronged mechanism involving the inhibition of TGF-β/Smad pathway activation, downregulation of tumor necrosis facto (TNF)-α and IL-1β, and reversal of EMT (Li et al., 2017; Wei et al., 2021). In addition, MSCs can correct inappropriate epithelial–mesenchyme relationships via paracrine secretion of stanniocalcin-1 (STC1), which alleviates oxidative and ER stress while suppressing TGF-β1 expression in AEII cells (Ono et al., 2015).

EV-mediated miRNAs transfer represents an important extension of MSC paracrine effects, as MSC-derived EVs modulate target gene expression through the delivery of specific miRNAs. For instance, BM-MSC-derived EVs attenuate IPF by delivering miR-186, which suppresses fibroblast activation via targeting the SOX4-DKK1 axis (Zhou et al., 2021). Human embryonic stem cell (ESC)-derived exosomes exert anti-inflammatory and antifibrotic effects through the delivery of miR-17–5p, which targets thrombospondin-2 (THBS2) (Liu et al., 2023). Furthermore, MSC-EVs inhibit myofibroblast differentiation by transferring miR-21 and miR-23, thereby downregulating TGF-β2 and its receptor TGF-βR2 within the TGF-β signaling cascade (Shi et al., 2021b). Conversely, the transfer of miR-29c and miR-129 by MSC-EVs promotes myofibroblast dedifferentiation, facilitating the resolution of established pulmonary fibrosis (Nataliya et al., 2023). Adoptive transfer of bone marrow monocytes preconditioned with human MSC-EVs also attenuates pulmonary inflammation and fibrosis in bleomycin-induced mouse models (Mansouri et al., 2019).

Recent studies have highlighted mitochondrial transfer and cytoprotection as critical therapeutic modalities. Promoting mitochondrial biogenesis in MSCs augments their capacity to shuttle functional mitochondria to injured lung cells, thereby restoring mitochondrial homeostasis, rescuing impaired mitophagy, and further attenuating pulmonary fibrosis progression (Huang et al., 2023). In addition, transplantation of adipose-derived MSCs (AD-MSCs) in a silicosis model ameliorated pulmonary fibrosis through anti-inflammatory and anti-apoptotic pathways, characterized by downregulated Caspase-3 expression and an increased Bcl-2/Bax ratio (Chen et al., 2018).

Notably, the intrinsic antifibrotic effect of MSCs is critically regulated by the local microenvironment. BM-MSCs isolated from IPF patients which exhibit a senescent phenotype, characterized by telomere attrition, mitochondrial dysfunction, accumulation of DNA damage, and impaired migratory and reparative capacities (Cárdenes et al., 2018). These senescent BM-MSCs can induce premature senescence in normal fibroblasts via paracrine signaling, thereby exacerbating bleomycin-induced pulmonary fibrosis. Thus, modulation of the inflammatory milieu by anti-fibrotic agents enhances the regenerative potential of MSCs. In addition, miR-199a-5p upregulation in IPF-derived MSCs exacerbates senescence via Sirt1/AMPK-mediated autophagy suppression; conversely, miR-199a-5p inhibition rescues this phenotype and augments their anti-fibrotic potential (Shi et al., 2021a).

Apart from MSCs, alternative stem cells have also exhibit promising antifibrotic effects. Distal airway stem cells (DASCs) leverage their robust regenerative capacity to integrate into injured lung tissue, differentiating into AE type I cells while attenuating fibrosis via concurrent suppression of collagen deposition and α-SMA expression (Shi et al., 2019). Similarly, airway basal stem cells (BSCs) promote regeneration but face survival challenges due to oxidative stress; however, pre-conditioning with CoQ10 mitigates H_2_O_2_-induced apoptosis and reactive oxide species (ROS) generation, thereby enhancing therapeutic efficacy in bleomycin-induced IPF models (Liu et al., 2022). Induced pluripotent stem cells (iPSCs) offer another avenue: their differentiated AEII-like cells can reverse established fibrosis by suppressing TGF-β and α-SMA expressions (Alvarez-Palomo et al., 2020). Furthermore, iPSC therapy can also regulate the Wnt signaling pathway, manifested by the downregulation of Wnt, β-catenin and Lef proteins, while upregulating the expression of Dickkopf-1 (Dkk1) (Bayati et al., 2023).

## 4 Clinical investigations of stem cell-based therapies for IPF: spotlight on safety and efficacy outcomes

### 4.1 MSCs

Clinical translation of stem cell-based therapy for F-ILD has been most widely studied in patients with IPF using MSCs as the stem cell type (Tzouvelekis et al., 2013; Chambers et al., 2014; Glassberg et al., 2017; Ntolios et al., 2018; Fishman et al., 2019; Averyanov et al., 2020; Campo et al., 2021). Table 1 summarizes the clinical trials of stem cell-based treatments for IPF and their published outcomes.

**TABLE 1:** Characteristics of the clinical trials evaluating stem cell-based therapies for IPF with published outcomes.

Study	Cell type/source	Patients	Dosage	Delivery mode	Study design	Outcomes
MSCs						
Tzouvelekis et al., 2013	Human ADSCs-SVF (autologous)	Mild-to-severe IPF (FVC >50% and DL_CO_ >35%; n = 14)	5 × 10^5^ cells/kg per infusion for a total of three infusions at monthly intervals	Intratracheal	Phase Ib, non-randomized, no placebo	No cases of serious or clinically meaningful TEAEs during 12-month and 24-month follow-ups
Ntolios et al., 2018	Human ADSCs-SVF (autologous)	Mild-to-severe IPF (80% > FVC >55% and DL_CO_ >35%; n = 14)	-	Intratracheal	Follow-up of above phase Ib study until time of death	FVC and 6MWT declined significantly at 24 months and DL_CO_ at 18 months; median OS and PFS in line with epidemiologic data
Chambers et al., 2014	Human PD-MSCs (allogeneic)	Moderately severe IPF (FVC ≥50% and DL_CO_ ≥ 25%; n = 8)	1 × 10^6^ vs. 2 × 10^6^ cells/kg	Intravenous	Phase Ib, non-randomized, no placebo	Only minor and transient acute adverse effects were reported; FVC, DLCO, 6MWT distance, and CT fibrosis score were unchanged at 6 months
Glassberg et al., 2017	Human BM-MSCs (allogeneic)	Mild-to-moderate IPF (FVC ≥50% and DL_CO_ ≥ 30%; n = 9)	2 × 10^7^, 1 × 10^8^, or 2 × 10^8^ cells per infusion	Intravenous	Phase I, non-randomized, no placebo (AETHER trial)	No serious TEAEs were reported; a 3.0% decline in mean FVC and 5.4% decline in mean DL_CO_ were observed at 60 weeks post-infusion
Fishman et al., 2019	Human BM-MSCs (allogeneic)	Mild-to-moderate IPF (FVC ≥50% and DL_CO_ ≥ 30%; n = 6)	2 × 10^7^ vs. 1 × 10^8^ cells per infusion	Intravenous	Data from AETHER trial to compare CT-evaluated changes in lung fibrosis	A slower progression of QLF and smaller decrease in DL_CO_ were observed in the 1 × 10^8^ cohort
Averyanov et al., 2020	Human BM-MSCs (allogeneic)	Rapidly progressing IPF (FVC ≥40% and DL_CO_ ≥ 20% with a decline in both of 10% over the previous 12 months; n = 20)	Two doses of 2 × 10^8^ cells every 3 months	Intravenous	Phase I/IIA, randomized, placebo-controlled	No significant TEAEs were reported during the 52-week follow-up; a significant improvement vs. placebo was initially observed for 6MWT distance at 13 weeks, DLCO at 26 weeks, and FVC at 39 weeks
Campo et al., 2021	Human BM-MSCs (autologous)	Mild-to-moderate IPF (FVC ≥50% and DL_CO_ ≥ 35%; n = 13)	Low (1 × 10^7^cells), intermediate (5 × 10^7^ cells), or high (1 × 10^8^ cells) dose	Intratracheal	Phase I, non-randomized, no placebo	No treatment-related severe AEs were observed; 8 of 13 patients suffered functional decline or clinical progression up to the end of follow-up
Li et al., 2025	Human UC-MSC-derived EVs plus routine treatment	IPF (n = 1), COPD with PF (n = 10), ILD (n = 7), post-inflammatory PF (n = 4), emphysema with PF (n = 2)	2 × 10^9^ particles	Nebulized	Phase I, randomized, placebo-controlled	No serious TEAEs were reported; significant improvements in FVC, MVV and respiratory health status
Lung progenitor cells/alveolar cells						
Serrano-Mollar et al., 2016	Human AEII cells (allogeneic)	Moderate and progressive IPF (FVC ≥50% and DL_CO_ ≥ 35%; n = 16)	Total number of cells was 1,000–1,200 ×10^6^	Intratracheal	Phase I, non-randomized, no placebo	No significant TEAEs were reported; no deterioration in pulmonary function, respiratory symptoms, or disease extent
Zhang et al., 2025	Human P63+ progenitor cells (autologous)	IPF (FVC ≥50% and DL_CO_ of 30%–79%; n = 16)	0.6 × 10^6^, 1 × 10^6^, 2 × 10^6^ or 3.3 × 10^6^ cells/kg	Intratracheal	Phase I, non-randomized, no placebo	No dose-limiting toxicity or cell therapy-related severe TEAEs were reported; significant improvements in lung gas transfer function and exercise ability and resolution of honeycomb lesions were observed in the higher dose cohort

Abbreviations: 6MWT, 6-min walk test; ADSC, adipose-derived stromal cell; AEII, alveolar epithelial type II; BM-MSCs, bone marrow-derived mesenchymal stem cells; COPD, chronic obstructive pulmonary disease; CT, computed tomography; DL_CO_; diffusion lung capacity for carbon monoxide; EV, extracellular vesicle; FVC, forced vital capacity; IPF, idiopathic pulmonary fibrosis; MSC, mesenchymal stromal/stem cell; MVV, maximal voluntary ventilation; PD-MSCs, placenta-derived mesenchymal stem cells; QLF, quantitative lung fibrosis; SVF, stromal vascular fraction; TEAE, treatment-emergent adverse event.

A core goal of the initial clinical studies was to assess the safety profiles of MSC-based therapies. A Phase Ib trial evaluated treatment-emergent adverse events (TEAEs) in 14 patients with mild-to-moderate IPF after three endobronchial infusions (at monthly intervals) of autologous adipose-derived stromal cells–stromal vascular fraction (ADSCs-SVF) at doses of 5 × 10^5^ cells per kg body weight per infusion (Tzouvelekis et al., 2013). Notably, there were no reports of infusion-related TEAEs during the 12-month period following the first infusion or ectopic tissue formation during a 24-month follow-up. Moreover, the median overall survival (OS) of 32 (range: 24–60) months and median progression-free survival (PFS) of 26 (range: 18–54) months were in accordance with published epidemiologic data (Ntolios et al., 2018), providing further evidence that ADSCs-SVF therapy has a favorable safety profile in patients with IPF. Another Phase 1 trial confirmed the safety and feasibility of administering autologous BM-MSCs to patients with mild-to-moderate IPF, with no serious TEAEs reported during the 12-month follow-up (Campo et al., 2021). In addition to the endobronchial route of delivery, intravenous administration of allogeneic stem cells has also demonstrated a favorable safety profile. For instance, the AETHER trial, which evaluated the safety of a single intravenous infusion of allogeneic BM-MSCs at various doses in nine patients with IPF, reported no serious TEAEs up to a dose of 2 ×10^8^ cells per infusion over a 60-week follow-up period (Glassberg et al., 2017). Similarly, a Phase Ib study showed that a single intravenous infusion of placenta-derived MSCs (PD-MSCs) in patients with mild-to-moderate IPF was well tolerated with no signs of worsening fibrosis during a 6-month follow-up (Chambers et al., 2014). Furthermore, a randomized, placebo-controlled, Phase I/IIA trial assessing the administration of a high cumulative dose of allogeneic BM-MSCs to patients with IPF and rapidly declining lung function detected no significant TEAEs compared to the placebo arm during a 52-week follow-up (Averyanov et al., 2020). These early studies collectively confirmed that MSCs of either allogeneic or autologous origin, administered through an intravenous or local endobronchial route, had an acceptable safety and tolerability profile in patients with IPF.

Given the encouraging preliminary safety profile of MSC therapy, some studies have evaluated the possible clinical benefits of this treatment. A Phase Ib study of patients with mild-to-moderate IPF administered PD-MSCs found that FVC, lung diffusing capacity for carbon monoxide (DL_CO_), 6-min walk test (6MWT) distance, and high-resolution computed tomography (HRCT)-assessed quantitative lung fibrosis (QLF) scores were unchanged over the course of a 6-month follow-up (Chambers et al., 2014). A quantitative HRCT-based analysis of patients in the AETHER trial evaluated the QLF score at baseline and at 24 and 48 weeks after the infusion of allogeneic BM-MSCs in patients with IPF (Fishman et al., 2019). Patients receiving the higher dose of BM-MSCs (1 × 10^8^ cells) exhibited slower disease progression over the 48-week infusion period than those receiving the lower dose (2 × 10^7^ cells), as evidenced by a smaller change from baseline in the QLF score (1.1% vs. 4.0%) and a smaller decline in DL_CO_ (2% vs. 17%). A more positive efficacy signal was reported in another placebo-controlled Phase IA/II study of patients with rapidly progressing IPF who received a higher cumulative dose of 1.6 × 10^9^ allogeneic BM-MSCs (Averyanov et al., 2020). When compared with placebo, treatment with BM-MSCs led to significant improvements in 6MWT distance from 13 weeks, DL_CO_ from 26 weeks, and FVC from 39 weeks. Notably, infusion of the maximal dose of 1.6 × 10^9^ MSCs was associated with increases in FVC (+3.7%) and 6MWT distance (+26.6%) relative to baseline at the 52-week follow-up, whereas decreases from baseline (−9.5% and −9.8%, respectively) were observed in the placebo group. The BM-MSC-treated group also exhibited a smaller decline in DL_CO_ from baseline compared to the placebo group (−5.1% vs. −12.9% at 52 weeks). Although this Phase IA/II trial found no significant between-group differences in mortality or HRCT-assessed QLF score, the notable improvements in FVC, 6MWT distance, and DL_CO_ provide preliminary yet clinically meaningful evidence that MSC-based therapy may slow the progression of IPF. In addition, a case report described a potential benefit of human umbilical cord-derived MSCs (UC-MSCs) in a patient with severe IPF who had required long-term oxygen therapy (Zhang et al., 2017). The physical performance, quality of life, and respiratory parameters of this patient were improved at the 12-month follow-up, and their requirement for oxygen therapy was reduced. Furthermore, nebulized delivery of human UC-MSC-derived EVs was considered to have an acceptable safety profile and promising effects on lung function indices in patients with various types of pulmonary fibrosis, including IPF, ILD, and fibrosis associated with chronic obstructive pulmonary disease (COPD) (Li et al., 2025).

Current clinical evidence indicates that MSCs may have the potential to stabilize or even improve lung function in patients with IPF, especially at higher doses. However, the vast majority of relevant data are derived from early-phase trials with small sample sizes, so the level of evidence is limited. Therefore, large-scale, randomized, double-blinded, placebo-controlled, Phase II/III clinical trials are needed to validate the safety and efficacy profiles of MSC-based therapies and optimize the cell sources, preparation techniques, and delivery regimens.

### 4.2 Transplantation of LPC and AEII cells

Although MSCs have received most attention in clinical translation studies, other cell types with regenerative potential have also been explored due to the limitations of MSC-based therapy such as low cell retention rate and heterogeneity (Lin et al., 2013). For instance, LPCs and AEII cells theoretically have better homing capacity and differentiation potential than MSCs, and the feasibility of their use in the treatment of IPF has been validated in Phase I clinical trials (Table 1).

A pioneering Phase I study assessed the safety and tolerability of intratracheal transplantation of allogeneic AEII cells for the treatment of moderate and progressive IPF (Serrano-Mollar et al., 2016). AEII cell transplantation was well tolerated during a 12-week follow-up, and there was an improvement in quality of life that manifested as a decrease in the severity of cough and dyspnea. Thirteen of the 16 patients (81.3%) remained in a stable condition after 12 months of follow-up, and their 6MWT distance was significantly increased compared with baseline, with a small decline in FVC (0.69%) and DL_CO_ (0.46%). A recent innovative study described the first-in-human administration of various doses (0.6 × 10^6^, 1 × 10^6^, 2 × 10^6^, or 3.3 × 10^6^ cells/kg) of autologous P63+ LPCs, which were cloned and expanded from bronchial biopsy specimens obtained from patients with IPF (Zhang et al., 2025). This open-label, dose-escalation, Phase I trial reported no dose-limiting toxicity or cell therapy-related severe TEAEs. Furthermore, significant improvements in lung gas transfer function and exercise ability, as well as resolution of honeycomb lesions, were observed in the higher dose cohorts (1 × 10^6^, 2 × 10^6^, and 3.3 × 10^6^ cells/kg bodyweight).

## 5 Beyond IPF: clinical investigations of stem cell-based therapy for other causes of F-ILD

### 5.1 CTD-ILD

Autologous HSCT is the most well-documented stem cell-based therapy for CTD-ILD, especially systemic sclerosis (SSc)-associated pulmonary fibrosis. Multiple RCTs have shown that HSCT yields significant improvements in skin flexibility, pulmonary function, and quality of life as well as long-term survival benefits in patients with progressive diffuse cutaneous SSc (dcSSc), compared to standard cyclophosphamide therapy (Burt et al., 2011; Van Laar et al., 2014; Sullivan et al., 2018). The ASSIST trial demonstrated that non-myeloablative autologous HSCT led to significant improvements in FVC and modified Rodnan skin score (mRSS), which were the primary endpoints (Burt et al., 2011). Although most published studies have utilized event-free survival, PFS or global assessment scores as the primary outcome, improvement in lung function has been widely evaluated as a key secondary endpoint (Van Laar et al., 2014; Sullivan et al., 2018; Henes et al., 2020). Since 2017, the updated European Alliance of Associations for Rheumatology (EULAR) recommendations have included HSCT as a treatment option in addition to high-intensity cyclophosphamide therapy for selected patients with early severe dcSSc, especially those without severe cardiorespiratory involvement (Kowal-Bielecka et al., 2017). A retrospective observational study of patients with SSc revealed a significant reduction in the total ILD extent score (5.1%; assessed by HRCT) and a significant increase in FVC (6.9%) 1 year after HSCT, whereas nonsignificant changes were observed with conventional cyclophosphamide therapy (Ciaffi et al., 2022). A real-world analysis also revealed a long-term improvement in the extent of ILD, with 78% of patients with SSc-ILD achieving a radiographic response at 24 months after HSCT (Pugnet et al., 2023). However, HSCT is associated with treatment-related toxicities, particularly infections and cardiac complications, as well as mortality (Burt et al., 2011; Van Laar et al., 2014; Sullivan et al., 2018). These drawbacks limit the wider application of HSCT, which is currently recommended for selected patients who do not respond well to conventional immunosuppressive therapy. Of course, the timing of transplantation is also critical, and there is a view that HSCT should be performed earlier in high-risk patients, in order to intervene before irreversible pulmonary fibrosis occurs, rather than be used as a salvage treatment option (Spierings et al., 2021).

The potential of HSCT to reset the immune system has been assessed in numerous investigations, whereas only a limited number of small-sample exploratory studies and case reports have evaluated whether MSC transplantation might exert a beneficial effect in CTD-ILD through a paracrine mechanism. One case report described a patient with SSc that had progressed to ILD despite therapy with mycophenolate mofetil and cyclophosphamide: subsequent treatment with PD-MSC EVs led to reductions in fibrotic changes, ground glass consolidation, and supplementary oxygen requirement, as well as improvements in respiratory symptoms (i.e., cough and dyspnea) and exercise tolerance (Assar et al., 2023). A recent review summarized the immunomodulatory and anti-fibrotic potential of AD-MSCs in CTD and the results of early small-sample human studies, which reported improvements in skin involvement, microcirculation, and lung function; however, supporting evidence from prospective RCTs remains scarce (Kotani et al., 2024). As our understanding of immune-fibrosis crosstalk in CTD-ILD, and particularly SSc-ILD, has deepened (Doskaliuk et al., 2025), novel cell-based therapies targeting specific cell types (e.g., CAR-T cells) or signaling pathways have been developed and are currently being investigated (Bautista-Sanchez and Khanna, 2024; Doskaliuk et al., 2025).

### 5.2 Post COVID-19 pulmonary fibrosis and long COVID

The COVID-19 pandemic has led to unprecedented attention being paid to pulmonary fibrosis and respiratory dysfunction after viral infection. Several clinical reports have investigated the safety and potential benefits of MSCs in the treatment of acute respiratory distress syndrome (ARDS) and other long COVID-19-related respiratory symptoms (Ji et al., 2020; Duong-Quy et al., 2025). For example, in five patients with persistent cough and dyspnea who were hospitalized for COVID-19 pneumonia, intravenous infusion of autologous ADSCs-SVF was associated with no significant TEAEs over a 12-month follow-up period (Carstens et al., 2024). Furthermore, four of these patients exhibited improvements in pulmonary function tests and CT-based findings. Another study of autologous AD-MSC infusion in 10 patients with post-COVID syndrome observed improvements in quality of life (Short Form-36 [SF-36] health survey), Fatigue Assessment Scale score, and symptom severity (visual analog scale), with no serious TEAEs reported (Vij et al., 2023). In addition, no serious TEAEs were observed in 10 patients with long COVID who received three infusions (at weekly intervals) of allogeneic human UC-MSCs, and the treatment was associated with repair of interstitial lung lesions and a gradual increase in the proportion of regulatory T cells (Tang et al., 2024). Similarly, a randomized, placebo-controlled trial involving patients with severe COVID-19 found that UC-MSC infusion improved quality of life and ameliorated lung damage over a 3-year follow-up period, and no long-term safety concerns or increased risks for tumorigenesis were observed (Yuan et al., 2025). These preliminary results suggest that MSCs from both allogeneic and autologous sources are well tolerated and show potential for the amelioration of postinfectious pulmonary fibrosis and long COVID symptoms. In addition to MSC-based therapy, a recently published randomized, placebo-controlled, Phase IIa trial demonstrated that infusions of allogeneic hematopoietic progenitor cells derived from cord blood were well tolerated in patients with post-COVID symptoms and produced significant and durable improvements in fatigue and quality of life (Huang et al., 2026).

### 5.3 Other causes of F-ILD

Individual case reports and preliminary studies have also described the use of stem cell therapy for F-ILD related to occupational or environmental exposures, such as drug- or radiation-induced F-ILD or, silicosis, although the evidence quality is relatively poor (Liu et al., 2015; Qiao et al., 2017; Chen et al., 2022; Saito et al., 2022). Preclinical research has shown that nebulized MSCs can alleviate acute lung injury and subsequent long-term pulmonary fibrosis after seawater drowning (Liu et al., 2024). Some rare immunodeficiency disorders also cause F-ILD, and clinical investigations have indicated that immune reconstitution with HSCT (rather than a therapy specifically targeting the ILD) is a potential curative treatment for these conditions. For instance, a case report described a child with X-linked hyper IgM syndrome who developed diffuse ILD that improved after anti-infective and immunoglobulin replacement therapy, and a complete cure was achieved through allogeneic HSCT (Li et al., 2022). The multi-target mechanisms of MSCs make them an attractive candidate for cell-based therapy of PPF, which encompasses a wide range of phenotypes across CTD-ILD and other F-ILDs. Targeted clinical trials are warranted to explore and validate the clinical benefits of MSCs in the treatment of these various disorders.

For some patients with F-ILD, the cause of death is related to underlying medical conditions such as COPD, pulmonary arterial hypertension, or pulmonary edema, and treatment of these complications also has a positive impact on quality of life and prognosis. A single-center study of patients with advanced COPD reported that intravenous infusion of autologous BM-MSCs was well tolerated and associated with an improvement in quality of life during a 12-month follow-up (Ribeiro-Paes et al., 2011). A multicenter, randomized, double-blind, placebo-controlled, Phase II trial with a 2-year follow-up randomly assigned 62 patients with moderate-to-severe COPD to either a group receiving four intravenous infusions of allogeneic BM-MSCs (1× 10^8^ cells per infusion) or a placebo group. (Weiss et al., 2013). The study found that MSC infusion was well tolerated (with no reports of infusion-related toxicity, death, or MSC-related serious adverse events [SAEs]) and exhibited potential systemic anti-inflammatory effects as evidenced by a significant reduction in the circulating C-reactive protein (CRP) level in patients with elevated baseline CRP. Preclinical studies using models of pulmonary emphysema have supported the potential of AD-MSCs to promote alveolar repair, inhibit cell apoptosis, and thereby improve lung function through paracrine mechanisms (Shigemura et al., 2006a; 2006b). There is also preclinical evidence for possible therapeutic effects of MSCs in other pulmonary diseases characterized by vascular remodeling and fibrosis, such as pulmonary arterial hypertension; these potential beneficial actions include an improvement in pulmonary vascular remodeling and hemodynamics through suppression of inflammation, regulation of apoptosis, and inhibition of endothelial-mesenchymal transition (De Mendonça et al., 2017).

## 6 Clinical considerations regarding stem cell product type and delivery routine

### 6.1 Stem cell sources and types: from whole-cell to cell-free therapies

Among the various types of stem cell evaluated as a potential therapy for F-ILD, MSCs have been most widely studied due to their ease of isolation from various tissues, low immunogenicity, and multi-lineage differentiation potential. Much research has focused on bone marrow-derived, umbilical cord-derived, and adipose-derived MSCs. Bone marrow was the main source of MSCs used in early clinical research, and the safety and preliminary efficacy of BM-MSCs has been demonstrated in IPF (Fishman et al., 2019; Averyanov et al., 2020), ARDS (Wilson et al., 2015), and COPD (Weiss et al., 2013). When compared with cells from adult sources, UC-MSCs have the advantages of ready availability, high proliferation ability, and low immunogenicity, and these cells have been evaluated as potential treatments for IPF (Zhang et al., 2017) and long COVID (Tang et al., 2024). In addition, a case report indicated that human UC-MSCs may confer clinical benefits in patients with anti-MDA5 antibody-positive dermatomyositis complicated by ILD despite treatment with conventional cyclophosphamide therapy (Zhou et al., 2025). A preclinical study found that human UC-MSCs exhibited superior antifibrotic and anti-inflammatory effects than BM-MSCs in a mouse model of bleomycin-induced pulmonary fibrosis, and BM-MSCs were also more effective at reversing lung injury, reducing body weight loss, and decreasing mortality (Ni et al., 2026). ADSCs/SVF are another option for MSC-based therapy that have the advantages of high cell yield and ready availability through minimally invasive surgery, and these cells have been assessed as treatments for IPF (Tzouvelekis et al., 2013; Ntolios et al., 2018) and long COVID (Vij et al., 2023; Carstens et al., 2024). Menstrual blood-derived MSCs (a relatively new source of MSCs) have also been explored as a potential therapy for severe COVID-19 due to their non-invasive method of acquisition and minimal ethical concerns (Heidari et al., 2024).

However, the therapeutic efficacy of MSCs from different sources may be affected by donor age (Alt et al., 2012), culture conditions (Samuelsson et al., 2009), and the disease microenvironment (Maffioli et al., 2017). Aging may reduce the proliferation and multi-directional differentiation potential of adipose-derived stromal cells (Alt et al., 2012), while obesity-related stress conditions can result in the loss of stemness markers for AD-MSCs, which is associated with a decline in regeneration ability (Al-Sammarraie et al., 2024). Proteomics analyses of human BM-MSCs have confirmed that MSCs exposed to proinflammatory factors secrete a large amount of immunomodulatory and angiogenesis-related proteins, suggesting that these cells may be more suitable for disease states when active inflammation is involved (Maffioli et al., 2017). Various clinical considerations should also be taken into account when selecting the most appropriate source of autologous or allogeneic MSCs. Although autologous MSCs theoretically avoid the risk of immune rejection and transmission of infectious disease, their quality and function may be affected by the long time required for their acquisition and preparation as well as the patient’s clinical status (i.e., age and comorbidities) (Perin et al., 2011; Alt et al., 2012). However, there is evidence that the properties of autologous AD-MSCs derived from patients with IPF do not differ from those derived from a healthy donor (Atanasova et al., 2022). Furthermore, a randomized trial that directly compared autologous and allogeneic BM-MSCs in patients with ischemic cardiomyopathy found that both had similar safety and efficacy profiles and that allogeneic cells did not elicit a notable donor-specific immune response, which provides a reference for the application of allogeneic MSCs in F-ILD (Hare et al., 2012).

PSC, including iPSC and ESCs, have an industrial advantage over MSCs because of their unlimited expansion capacity, and preclinical studies using models of F-ILD have reported potent antifibrotic effects of iPSCs (Banerjee et al., 2012; Chakraborty et al., 2024; Nakamura et al., 2024). Somatic stem cells derived from lung tissue, such as lung spheroid cells or AEII cells, theoretically have intrinsic lung regeneration properties. STIM-activating enhancer-positive exosomes derived from AEII cells have been reported to reduce pulmonary fibrosis and mortality in a mouse model of bleomycin-induced fibrosis through a unique mechanism that regulates the metabolic reprogramming of tissue-resident alveolar macrophages (Feng et al., 2023). Preclinical studies have provided evidence that, when delivered by inhalation, autologous or allogeneic lung spheroid cells (along with their secretomes and exosomes) are safe, exhibit therapeutic potential in pulmonary fibrosis models, and may be superior to their MSC counterparts in promoting lung repair (Cores et al., 2017; Dinh et al., 2020). Clinical application of autologous LPCs and allogeneic AEII cells has been explored in patients with progressive IPF (Serrano-Mollar et al., 2016; Zhang et al., 2025), representing an important advance toward individualized regenerative therapy using the patient’s own LPCs.

In recent years, there has been increasing interest in stem cell-derived EVs, especially exosomes, as a “cell-free” therapeutic strategy. This approach could theoretically avoid the potential risks of live cell transplantation, such as pulmonary embolism, abnormal cell retention, and tumor formation, and nebulized EVs prepared from allogeneic AD-MSCs have been shown to be well tolerated in healthy subjects (Shi M. et al., 2021). More importantly, a Phase 1 trial has explored the safety and efficacy of nebulized human UC-MSC-derived EVs in patients with pulmonary fibrosis (Li et al., 2025). Patients receiving UC-MSC-EVs plus routine treatment exhibited significant improvements in lung function (as assessed by FVC and maximal voluntary ventilation), and fibrosis regression on CT imaging was even observed in some patients with advanced disease. Additionally, PD-MSC-derived EVs have produced beneficial effects (based on symptom improvement and imaging findings) in patients with SSc-ILD (Assar et al., 2023). However, the comparison of therapeutic potentials for EVs derived from different MSC sources remains unclear. Moreover, MSC-derived conditioned medium therapy is also being explored as an alternative cell-free therapy. The feasibility of using amniotic fluid-derived EVs, rather than MSC-derived EVs, has also been examined, with one case report suggesting that administration of these EVs to a patient with long COVID was safe and potentially led to improvements in respiratory symptoms and oxygenation (Mitrani et al., 2021). Investigations to improve the targeting and efficacy of stem cells have also been conducted. For example, miR-146a-5p-enriched EVs were reported to produce enhanced anti-inflammatory effects (Wang et al., 2026), while bioenzymatic single-cell microencapsulation of MSCs was found to promote their retention and survival in a model of pulmonary fibrosis (Xuan et al., 2026). Although assessments of outcomes in human clinical trials are still in the early stages, cell-free therapy is considered to have great potential for clinical translation due to its safety profile and scope for industrial scaling.

### 6.2 Intravenous, intratracheal, and nebulized delivery

The delivery route has important effects on the *in vivo* distribution, bioavailability, targeting, therapeutical window, and safety profiles of stem cells (Moodley et al., 2011). Intravenous infusion is the most widely used and data-rich route in clinical studies because this method of administration is relatively simple to perform, standardize, and repeat. Intravenous administration of MSCs has exhibited acceptable safety properties in patients with IPF, either when given as a single infusion (Glassberg et al., 2017) or multiple high-dose infusions (Averyanov et al., 2020). However, the disadvantages of intravenous stem cell infusion include a substantial first-pass effect in the lung and retention of MSCs in the liver, spleen, or other non-target organs, which can limit the clinical effect in pulmonary fibrotic lesions and increase the risk of systemic exposure (Barbash et al., 2003). Direct administration of cells to the airway by intratracheal or nebulized delivery have been evaluated as methods to overcome the limitations of intravenous infusion and improve lung targeting, and there is clinical proof-of-concept for the use of these administration routes in patients with IPF (Tzouvelekis et al., 2013; Li et al., 2025) or other respiratory diseases accompanied by pulmonary fibrosis (Chang et al., 2014; Dominguez-Pinilla et al., 2025). However, repeated application of transbronchial perfusion is limited by its invasiveness, and nebulized delivery may be more suitable for cell-free therapeutic agents such as exosomes and conditioned media (Gao et al., 2026). The construction of a liposome-exosome hybrid delivery system has been reported to enhance the targeting of pulmonary fibrosis (Wang et al., 2024). In addition, a preclinical study utilizing a model of fibrosis in the aged lung found that intravenous administration delivered EVs more effectively to normal and fibrotic lung tissues than intrapleural or intratracheal administration, but therapeutic effects were only observed when the EVs were administered before the development of fibrosis (Amtil-Ouahdi et al., 2025). Taken together, the above findings suggest that the delivery route may need to be optimized according to the F-ILD stage, the stem cell-based agent being used, and the individual patient’s characteristics (including age); these issues will require further research in future investigations.

## 7 Challenges and future directions for clinical translation and clinical trial design

Pilot clinical studies have reported promising outcomes for stem cell-based therapy in patients with various forms of F-ILD, ranging from IPF (Averyanov et al., 2020; Li et al., 2025; Zhang et al., 2025) to CTD-ILD (Spierings et al., 2021) and even long COVID syndrome (Tang et al., 2024; Yuan et al., 2025). Furthermore, autologous HSCT has an established role as a disease-modifying treatment in high-risk patients with progressive SSc-ILD (Sieiro Santos and Del Galdo, 2025). However, translating stem cell-based therapy into routine clinical practice still faces numerous challenges.

The first issue that needs to be addressed is the standardization and optimization of stem cell-based therapeutic strategies. The minimal definition criteria for MSCs proposed by the International Society for Cellular Therapy (Dominici et al., 2006) and the International Federation for Adipose Therapeutics and Science/International Society for Cellular Therapy joint statement regarding MSCs/SVF (Bourin et al., 2013) have provided a basic framework for the quality control of stem cell products. However, the preparation techniques and amplification conditions (including serum concentration and inoculation density) vary among different laboratories and clinical centers, which may substantially affect the safety profiles and immunomodulatory potential of stem cells *in vivo*. Therefore, more unified and reproducible manufacturing practices and quality control specifications should be established to ensure batch-to-batch consistency in stem cell function and behavior, as this will facilitate inter-study comparisons of clinical outcomes. Recent insights into apoptotic MSC-mediated immunomodulation have profound implications for MSC product quality assurance. Of particular note, He et al. proposed that apoptotic MSCs exert potent immunomodulatory effects even at low doses (∼5 × 10^4^ cells), achieving outcomes equivalent to those of high-dose viable MSCs (1 × 10^6^ cells) (He et al., 2021). Mechanistically, this effect is mediated by the exposure of phosphatidylserine on apoptotic cells, which modulates immune responses of the local immune-microenvironment through Mer tyrosine kinase (MerTK)- and C–C motif chemokine receptor 2 (CCR2-dependent pathways). These insights challenge the prevailing reliance on cell viability as the primary release criterion in current Good Manufacturing Practice (cGMP) settings. Consequently, there is an emerging need to expand future product specification protocols to include the quantitative characterization of apoptotic fractions and their associated immunomodulatory potential, notably through PS/MerTK-dependent potency assays. Conversely, it is imperative to delineate the optimal threshold of apoptotic cells and their safety boundaries, specifically addressing risks associated with excessive apoptosis, necrosis, *in vivo* fate, and overall biosafety.

Treatment regimens also need to be optimized, including determination of the optimal stem cell product (i.e., MSCs from different sources, LPS or EVs) and the delivery route (intravenous, intratracheal or nebulized), the appropriate dosage and frequency, as well as the favorable therapeutic time windows. In response to the aforementioned challenges, a series of optimization strategies are being explored. For instance, clustered regularly interspaced short palindromic repeats (CRISPR)/CRISPR-associated protein 9 (Cas9) engineering is being employed to enhance the homing capacity and anti-apoptotic properties of stem cells or induce the production of secretomes (Hazrati et al., 2022). Improvements in delivery route include the development of a compound nanoparticle system for nebulized delivery and a microgel encapsulation technology that improve pulmonary bioavailability and prolong residence time while minimizing systemic adverse effects (Chang et al., 2020; Gao et al., 2026; Xuan et al., 2026).

Given the heterogeneity of F-ILD, the patient subgroups most likely to benefit from stem cell-based therapy are being identified according to etiology (such as idiopathic, autoimmune, or postinfection), molecular phenotype, disease stage, and immune characteristics. For example, patients with IPF often exhibit disease-associated changes in peripheral blood immune phenotypes (Atanasova et al., 2022), and the identification of biomarkers that predict treatment responses will facilitate a move towards personalized medicine. Interestingly, the miRNA profile of sputum-derived exosomes differs between patients with IPF and healthy individuals (Njock et al., 2019). Moreover, the miR-142–3p level is negatively correlated with DL_CO_, suggesting that this could serve as a potential biomarker for disease severity. Furthermore, biomarkers can not only facilitate the screening of patients for potential responders but also act as exploratory endpoints, enabling early detection of the intervention’s impact on the molecular phenotype of the disease before significant structural or functional changes occur. Even IPF exhibits different clinical stages, hence precisely defining the disease progression phenotype will be crucial for stratifying patients according to their predicted response to stem cell therapy (Inoue et al., 2020). Another advance would be the establishment of *in vitro* models that are closer to the pathological condition *in vivo*, such as patient-specific induced PSC-derived alveolar organoids, which can be employed for mechanism studies, drug testing, and personalized treatment prediction (Kim et al., 2021; Cao et al., 2025).

There are also unique challenges in the design of definitive Phase II/III clinical trials of stem cell-based therapy. First, most of the positive evidence obtained to date comes from Phase I/II trials with a follow-up period of 1–2 years, which have not observed any instances of fatal SAEs related to intravenous or local infusion of MSCs or EVs (Glassberg et al., 2017; Averyanov et al., 2020; Li et al., 2025). However, long-term safety profiles are lacking, which are crucial for the assessment of potential tumorigenicity and the risk of ectopic tissue formation. Second, in addition to the conventional efficacy endpoints (such as FVC, DL_CO_, 6MWT, and imaging scores), more sensitive indicators should be introduced such as quantitative CT fibrosis analysis (Fishman et al., 2019), biomarkers that predict treatment response (e.g., specific immune cell subsets, circulating factors, or imaging features) (Atanasova et al., 2022; Broens et al., 2022), and comprehensive scores reflecting quality of life. Third, clinical trials should include more representative populations to allow generalization of the findings including clinical outcomes (Saltzman et al., 2021). Fourth, given that anti-fibrotic drugs have become the standard treatment for F-ILD, future trial designs need to consider the combined or sequential application of stem cell therapy with existing standard treatments and clearly define whether the role of therapy is disease modification, symptom relief, or improvement of quality of life. Finally, the cost-effectiveness and accessibility of stem cell-based therapies are practical issues that must be taken into account.

Future studies on stem cell-based therapies for F-ILD should be interpreted within the rapidly evolving therapeutic landscape of IPF/PPF. Recent literature has emphasized that the management of IPF/PPF is moving beyond the two approved antifibrotic agents toward multi-target, combination, and precision-medicine approaches, with emerging pharmacological pathways such as preferential phosphodiesterase-4B (PDE4B) inhibition, integrin αvβ6, prostacyclin-cAMP signaling, lysophosphatidic acid (LPA) pathway, Janus kinase (JAK)-STAT signaling, Wnt/β-catenin, TGF-β, and senescence-directed therapies (Giulianelli et al., 2025; Kondoh et al., 2025; Carriera et al., 2026). Concurrently, novel agents including nerandomilast (a PDE4B-selective inhibitor) (Maher et al., 2025; Richeldi et al., 2025), admilparant (an LPA1 antagonist) (Corte et al., 2025; Kreuter et al., 2025), and inhaled treprostinil (a PDE-5 inhibitor) (Nathan et al., 2022), exemplify the ongoing shift toward mechanism-informed, phenotype-targeted therapeutic strategies for PPF/IPF. In this context, stem cell–based therapies should not be framed as alternatives to antifibrotic agents, but more on whether it can be integrated into precision and combination treatment frameworks. This issue is especially relevant the given the profound biological heterogeneity of F-ILD and the systemic shift in clinical management paradigms toward precision interventions guided by definable phenotypes (e.g., progressive fibrotic phenotype). However, combination therapeutic strategies necessitate rigorous assessment of additive toxicity, tolerability, and optimal sequencing relative to antifibrotic or immunomodulatory agents. Combinational or sequential treatment strategies involving the co-administration of stem cells or exosomes with existing anti-fibrotic drugs (such as pirfenidone or nintedanib) are also being explored, as synergistic effects have been reported in rat models of pulmonary fibrosis (Hassan et al., 2025; Abd Elhamid et al., 2026). Critically, however, the current evidence base remains predominantly preclinical or confined to early-phase clinical trials, with safety established as the primary endpoint, with robust efficacy data still lacking. Moreover, future clinical trials of stem cell-based therapies should transcend conventional single-endpoint metrics and adopt multidimensional outcome assessments that comprehensively capture longitudinal changes in lung function, patient-reported symptoms, quantitative imaging changes, and major safety concerns.

In the context of F-ILD treatment, the comparative and integrative potential of therapeutic vaccines and MSCs therapy warrants further exploration. Therapeutic vaccines facilitate active immunomodulation by targeting fibrosis-associated antigens or pathogenic immune cells, thereby inducing antigen-specific tolerance or regulatory immune responses. Subtypes of F-ILD characterized by distinct immune-driven pathogenesis are likely better suited for therapeutic vaccination, whereas fibrosis driven by epithelial injury and regenerative failure may be more amenable to MSCs therapy. For autoimmune disorders such as SSc, immunotherapeutic approaches utilizing vaccines to target B cells or key cytokines remain in the preclinical and early-phase clinical stages of evaluation (Doskaliuk et al., 2025; Sieiro Santos and Del Galdo, 2025). In addition, therapeutic vaccines necessitate patient-specific customization based on individual antigen profiles, whereas MSC therapies can be developed as universal, off-the-shelf products. Future investigations will prioritize the exploration of sequential therapeutic paradigms, that initially administering MSCs to attenuate inflammation and facilitate tissue repair, followed by therapeutic vaccination to sustain immune tolerance, or combinatorial approaches designed to elicit synergistic efficacy.

## 8 Summary and prospects

In conclusion, the use of stem cells and their derivatives in the treatment of F-ILD has moved from a distant vision to the initial stage of clinical validation. Therapeutic strategies based on MSCs and their derivatives (particularly EVs) have demonstrated an acceptable safety profile and potential therapeutic benefits in early clinical trials involving patients with F-ILD such as IPF and SSc-ILD. Autologous HSCT has established its status as a disease-modifying therapy in refractory and rapidly progressive SSc. Different administration routes have been explored, with non-invasive targeted delivery by nebulization exhibiting unique advantages. However, stem cell-based therapy is still in the early stage of clinical translation. Heterogeneity in the therapeutic effects of stem cell therapies, a lack of long-term safety data, the complex mechanisms of action, and issues relating to standardized manufacturing processes are the main challenges currently being faced.

Future work should position stem cell-based therapy within the broader evolution of IPF/PPF treatment and focus on that following key directions: 1) The design of multicenter, large-sample, randomized, controlled, Phase II/III clinical trials should align with contemporary IPF/PPF studies, incorporating composite endpoints, longitudinal lung function trajectories, acute exacerbations, hospitalizations, mortality, patient-reported outcomes, and long-term safety; 2) Cell-free strategies, including MSC-derived EVs, engineered cells, and biomaterial-assisted delivery, warrant further investigation to enhance their targeting ability and efficacy; 3) The establishment of standardized manufacturing practices/quality control specifications for stem cells and EVs, such as optimal dose, route of administration, timing, cell heterogeneity, and batch-to-batch consistency; 4) The development of new technologies to achieve targeted pulmonary delivery of stem cells or EVs, thereby increasing the local concentration and reducing systemic exposure; 5) Evaluation of whether the combined or sequential application of stem cell therapy (or EVs) with existing anti-fibrotic drugs (nintedanib/pirfenidone) or immunosuppressants produces synergistic effects; and 6) Utilization of single-cell sequencing, proteomics, and other technologies to identify biomarkers that predict treatment responses, towards biomarker-guided patient selection and PPF-oriented phenotypes, rather than assuming a single therapeutic niche.
